# Multi institutional quantitative phantom study of yttrium-90 PET in PET/MRI: the MR-QUEST study

**DOI:** 10.1186/s40658-018-0206-y

**Published:** 2018-04-04

**Authors:** Nichole M. Maughan, Mootaz Eldib, David Faul, Maurizio Conti, Mattijs Elschot, Karin Knešaurek, Francesca Leek, David Townsend, Frank P. DiFilippo, Kimberly Jackson, Stephan G. Nekolla, Mathias Lukas, Michael Tapner, Parag J. Parikh, Richard Laforest

**Affiliations:** 1 Department of Radiation Oncology, Washington University School of Medicine, 4921 Parkview Place, Campus Box 8224, St. Louis, MO 63110 USA; 2 Translational and Molecular Imaging Institute, Icahn School of Medicine at Mount Sinai, 1470 Madison Ave, New York, NY 10029 USA; 3 Department of Biomedical Engineering, City College of New York, 160 Convent Ave, New York, NY 10031 USA; 4 Siemens Healthineers, Siemens Medical Solutions USA, Inc., 40 Liberty Boulevard, Malvern, PA 19355-9998 USA; 5 Molecular Imaging, Siemens Healthineers, 810 Innovation Dr, Knoxville, TN 37932 USA; 6 Department of Circulation and Medical Imaging, Faculty of Medicine and Health Sciences, NTNU, Norwegian University of Science and Technology, Postboks 8905, 7491 Trondheim, Norway; 7 Department of Radiology, Icahn School of Medicine at Mt. Sinai, One G. Levy Pl., Box 1141, New York, NY 10029 USA; 8 Agency for Science Technology and Research, National University of Singapore Clinical Imaging Research Centre, 14 Medical Drive, #B1-01, Singapore, 117599 Singapore; 9 Department of Nuclear Medicine, Cleveland Clinic, Mail Code Jb3, 9500 Euclid Ave, Cleveland, OH 44195 USA; 10 NYU Medical Center, 333 E 38th St, New York, NY 10016 USA; 11 Klinik und Poliklinik für Nuklearmedizin, TU München, Strasse 22, 81675, Munich, Germany; 12 Department of Nuclear Medicine, Charité—Universitätsmedizin Berlin, Charitépl. 1, 10117 Berlin, Germany; 13 Siemens Healthcare GmbH, Berlin, Germany; 14 Sirtex Medical Ltd, Level 33, 101 Miller St, North Sydney, NSW 2060 Australia; 15 ABX-CRO Advanced Pharmaceutical Services, 1 Begonia Road, Normanhurst, NSW 2076 Australia; 16 Mallinckrodt Institute of Radiology, Washington University School of Medicine, 510 S. Kingshighway, Campus Box 8225, St. Louis, MO 63110 USA

**Keywords:** Yttrium-90, PET, PET/MRI, Radioembolization

## Abstract

**Background:**

Yttrium-90 (^90^Y) radioembolization involves the intra-arterial delivery of radioactive microspheres to treat hepatic malignancies. Though this therapy involves careful pre-treatment planning and imaging, little is known about the precise location of the microspheres once they are administered. Recently, there has been growing interest post-radioembolization imaging using positron-emission tomography (PET) for quantitative dosimetry and identifying lesions that may benefit from additional salvage therapy. In this study, we aim to measure the inter-center variability of ^90^Y PET measurements as measured on PET/MRI in preparation for a multi-institutional prospective phase I/II clinical trial.

Eight institutions participated in this study and followed a standardized phantom filling and imaging protocol. The NEMA NU2-2012 body phantom was filled with 3 GBq of ^90^Y chloride solution. The phantom was imaged for 30 min in listmode on a Siemens Biograph mMR non-TOF PET/MRI scanner at five time points across 10 days (0.3–3.0 GBq). Raw PET data were sent to a central site for image reconstruction and data analysis. Images were reconstructed with optimal parameters determined from a previous study. Volumes of interest (VOIs) matching the known sphere diameters were drawn on the vendor-provided attenuation map and propagated to the PET images. Recovery coefficients (RCs) and coefficient of variation of the RCs (COV) were calculated from these VOIs for each sphere size and activity level.

**Results:**

Mean RCs ranged from 14.5 to 75.4%, with the lowest mean RC coming from the smallest sphere (10 mm) on the last day of imaging (0.16 MBq/ml) and the highest mean RC coming from the largest sphere (37 mm) on the first day of imaging (2.16 MBq/ml). The smaller spheres tended to exhibit higher COVs. In contrast, the larger spheres tended to exhibit lower COVs. COVs from the 37 mm sphere were < 25.3% in all scans. For scans with ≥ 0.60 MBq/ml, COVs were ≤ 25% in spheres ≥ 22 mm. However, for all other spheres sizes and activity levels, COVs were usually > 25%.

**Conclusions:**

Post-radioembolization dosimetry of lesions or other VOIs ≥ 22 mm in diameter can be consistently obtained (< 25% variability) at a multi-institutional level using PET/MRI for any clinically significant activity for ^90^Y radioembolization.

## Background

Yttrium-90 (^90^Y) radioembolization involves the intra-arterial delivery of radioactive microspheres to primary or metastatic disease in the liver. ^90^Y decays primarily with β^−^ emission (mean 0.937 MeV, 64.2 h half-life, 2.5 mm mean soft tissue penetration, 11 mm max tissue penetration) [1], allowing for high amount of radiation dose within a well-confined region. The current commercially available microspheres, TheraSpheres (glass microspheres; BTG, London, UK) and SIR-Spheres (resin microspheres; Sirtex Medical, Sydney, Australia), were first approved in the USA by the Food and Drug Administration in 1999 and 2002 and received the European CE mark in 2005 and 2002, respectively [2, 3]. Since then, ^90^Y microsphere radioembolization has continued to grow as a third-line therapy for patients with some clinical trials into using this technology as a first-line therapy concurrent with FOLFOX-based chemotherapy [4]. While the use of ^90^Y microspheres to treat cancer in the liver has been utilized for over 50 years [5], the treatment planning protocol from pre-treatment imaging to prescription activity calculation is not well optimized. Standard protocol does not allow for assessing the actual distribution of the microspheres or for predicting the effectiveness of treatment or possible toxicity events without waiting for follow-up imaging studies at least 30 days after treatment [6]. Patients who undergo ^90^Y radioembolization typically have poor prognoses and earlier assessment of therapy immediately after injection could guide optimization of therapy.

Recently, there has been an interest in performing post-radioembolization imaging and dosimetry to assess the microsphere distribution and predict clinical outcomes. Early work involved Bremsstrahlung SPECT, utilizing the high Bremsstrahlung flux from the β^−^ emission of ^90^Y [7–9]. However, these images suffer from poor spatial resolution, making it difficult to identify small areas of uptake and the heterogeneous distribution of the microspheres [10, 11] as well as perform quantitative evaluation. Early case studies found that the small positron yield (0.0032%) of ^90^Y was sufficient for imaging the microsphere distribution by PET [12–14]. Since then, several single-center studies have reported the ability to predict clinical outcomes using dosimetry derived from ^90^Y PET images following radioembolization in both primary liver cancer [15] and metastatic colorectal cancer [16–18].

Multi-center clinical studies with quantitative end points, typically involves imaging a standard phantom to test quantitative accuracy and inter-center variability. Fahey et al. evaluated inter-center reproducibility and variability in a multi-center study involving fluorine-18 (^18^F) on PET/CT [19]. Another similar study was performed by Willowson et al. in the multi-center QUEST phantom study for measuring inter-site variability of ^90^Y on PET/CT [20].

The purpose of this study is to measure both the inter- and intra-center variability of quantitatively measuring ^90^Y on PET/MR in preparation for a multi-center phase I/II clinical trial (NCT02611661). Our study mirrors the work that was performed by Willowson et al. in the QUEST study but, due to the difference in scanner designs and electronics, focuses instead on PET/MRI scanners. Furthermore, this study uses the same PET/MRI system model and identical reconstruction parameters across all centers, rather than allowing centers to reconstruction their own data with their own parameters. This was to test the true performance of the scanner with ^90^Y without extra uncertainty introduced by varying choices in reconstruction parameters.

## Methods

A total of eight institutions across four countries participated in this phantom study. All sites followed a strict protocol for both filling and imaging the phantom. Three NEMA NU2-2012 image quality (IQ) body phantoms (PTW Freiburg GmbH, Freiburg, Germany) with vendor-provided foam cradles and positioning devices were shared between the eight institutions.

### Phantom preparation

Before filling the phantom with activity, the volume of the background compartment was measured by its water weight.

3.6 GBq in 1.4 mL of ^90^Y chloride solution (PerkinElmer, Waltham, MA) was shipped to each institution. The supplier’s calibrated activity listed on the shipping document was used as the “ground truth” for the activity within the vial. Each site recorded the amount of activity reported by their department’s dose calibrator for comparison against the reported amount on the shipping label. The manufacturer and model of each site’s dose calibrator is listed in Table 1.

**Table 1:** Dose calibrator and settings used at each site to measure ^90^Y activity and verify against that reported on shipping label

Site number	Dose calibrator manufacturer	Model	Setting for ^90^Y
1	Capintec	CRC-12	Channel 53 × 10
2	Capintec	CRC-55 T PET	Channel 010
3	Capintec	CRC-15R	Channel 48 × 10
4	Capintec	CRC-15R	Channel 56 × 10
5	Capintec	55 t W	Channel 48 × 10
6	Veenstra Instruments	VIK 2025051	Factor 902, scale 100
7	MED	ISOMED 2010	Factor 2.3 (geometry: 3 ml syringe/1.4 ml solution)
8	Capintec	CRC-15R	Channel 48 × 10

The entire contents of the vial were completely emptied into 1300 mL of water with 100 mg of either DTPA or EDTA added to prevent binding of ^90^Y chloride to the walls of the phantom. Activity was drawn from this solution to fill the six spheres (diameters 37, 28, 22, 17, 13, and 10 mm). Once all spheres were filled, the remaining solution was emptied into the background compartment of the NEMA IQ phantom, and the remaining volume of the background compartment was filled with water. This resulted in an approximately 8:1 sphere-to-background activity concentration ratio. The center lung insert for the phantom was a solid structure made of polyethylene material. The total recorded activity in the phantom was the total activity listed on the shipping document minus any residual activity in the vial and syringe.

### Image acquisition and reconstruction

The phantom was imaged at five time points (day 0, day 3, day 5, day 7, and day 10) at each institution on Siemens Biograph mMR non-time-of-flight (non-TOF) PET/MRI scanners, representing a range of total activities from 0.3 to 3.0 GBq. These values correspond to the full range of activities administered to patients treated with resin microspheres. The phantom was positioned in a foam cradle with an accompanying positioning device, as described by Ziegler et al. [21], to center the phantom in the field of view (FOV) and allow for reproducible placement of the phantom between scans and institutions. The phantom was imaged for 30 min in listmode acquisition in a single station. Due to the longer axial FOV of the Biograph mMR (25.6 cm), we are able to reduce the number of stations compared to other scanners with a shorter FOV (e.g., 15–16 cm), which typically require a minimum of two stations [20]. For post-radioembolization patients at our clinic (WUSM), we acquire PET data in a single station. At one of the sites, three back-to-back PET acquisitions (same phantom filling) were performed to evaluate intra-center variability. Another site (Washington University, St. Louis, MO) repeated the phantom filling with 54.4 MBq fluorine-18 (^18^F) and imaged at a single time point (15 min listmode acquisition) for comparison to a high statistic dataset. This ^18^F dataset was also reconstructed into ten smaller datasets, consisting of 3 s each of listmode data, to contain a similar amount of trues statistics to that seen in the ^90^Y PET data. Those same ten data sets were reconstructed with additional randoms added to the sinograms to obtain a similar randoms fraction as that observed in the ^90^Y PET data. A detailed description of these methods are described in [22]. This was to compare measurements from ^18^F data containing a similar amount of trues count statistics as that exhibited in ^90^Y PET data on the highest activity day of imaging.

All raw PET data were sent via a secure data server (ABX-CRO Advanced Pharmaceutical Services, Dresden, Germany) to a central site (Washington University, St. Louis, MO) for reconstruction and analysis by a single investigator (N.M.). All PET reconstructions were performed using e7tools, the offline research reconstruction software provided by Siemens, with a vendor-provided, CT-derived attenuation map of the NEMA 2007/IEC 2008 Body phantom for attenuation correction, as described by Ziegler et al. [21] The attenuation map was manually inspected and registered for each PET data set since they were not measured directly from the PET/MRI scanner. Images were reconstructed with 3D ordinary Poisson ordered subset expectation maximization (OP-OSEM), with the following parameters determined from a previous phantom study: 3 iterations, 21 subsets, 5 mm full-width-half-maximum Gaussian post-reconstruction filter, point spread function (PSF) compensation, and using absolute scatter correction with 4.17 × 4.17 × 2.03 mm^3^ voxels [23].

### Image post-processing and analysis

**Fig. 1:**
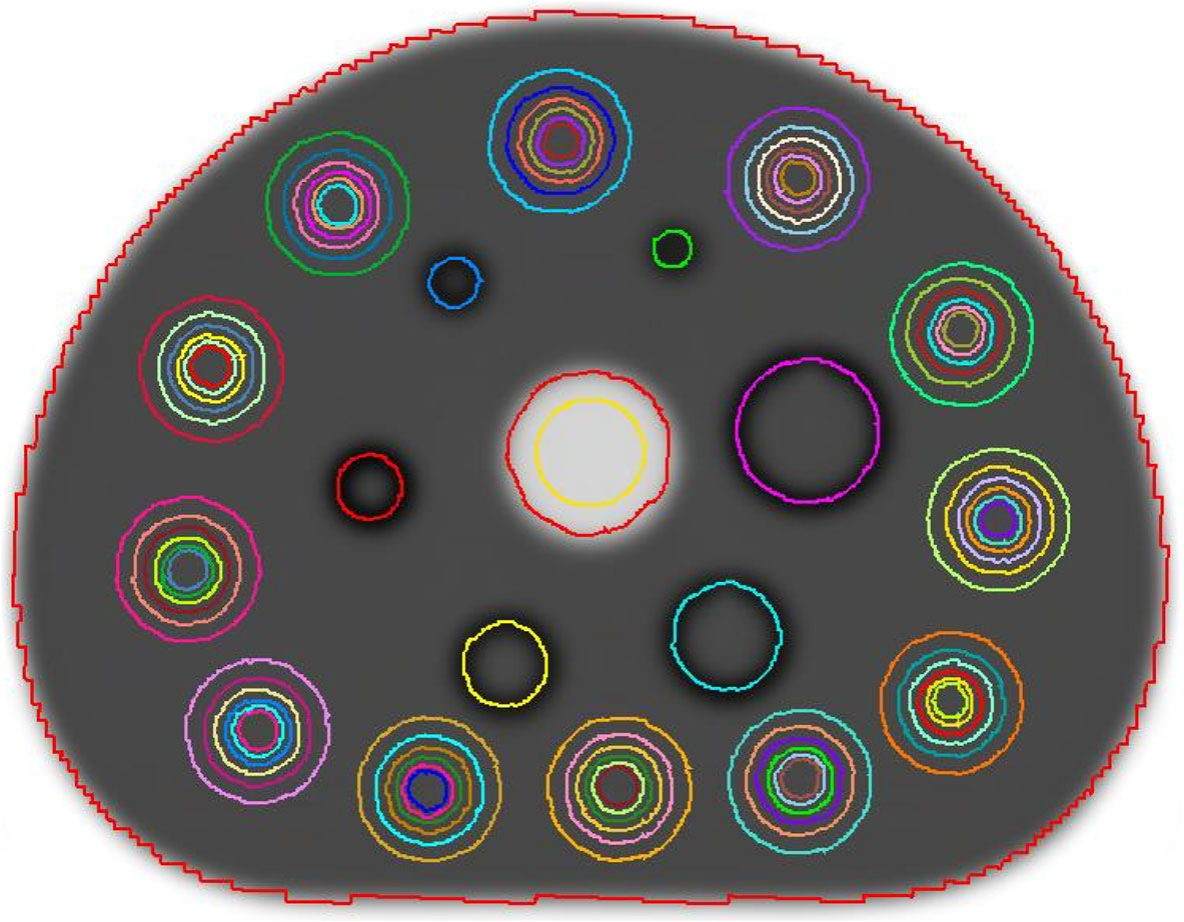
Attenuation map of NEMA 2007/IEC 2008 body phantom with VOIs drawn around each of the six fillable spheres, 72 VOIs drawn in the background compartment, and a 28-mm diameter VOI drawn in the cold insert (center, yellow)

All image post-processing, including drawing volumes of interest (VOIs) and extracting statistics from those VOIs, was performed in MIM v6.6.7 (MIM Software, Cleveland, OH). VOIs were drawn on the attenuation map of the phantom (see Fig. 1). The VOIs on the spheres were drawn as spherical VOIs with a diameter matching the known sphere diameter. The VOIs with their corresponding purpose for quantitative assessment, according to NEMA NU 2-2007 guidelines, are summarized in Table 2. Each PET image was fused to the corresponding attenuation map for transfer of VOIs.

**Table 2:** Quantities for volume of interest (VOI) analysis

VOI and size	Measured quantity	Purpose
Field of view (FOV)	Total activity (GBq)	Accuracy in total injected activity measurement
Fillable spheres: 37, 28, 22, 17, 13, 10 mm	Recovery coefficient of mean activity concentration (MBq/ml)	Accuracy in VOI measurements from partial volume effects
Background spheres: 12 of 37-mm diameters	Recovery coefficient of mean activity concentration (MBq/ml)	Accuracy in warm background VOI measurements
Background spheres: 12 each of 37-, 28-, 22-, 17-, 13-, 10-mm diameters	Standard deviation of mean activity concentration (MBq/ml)	Variability in warm background measurements (noise)
Insert: 28-mm diameter, 160-mm length	Mean activity concentration (MBq/ml)	Background counts

The recovery coefficients (RCs) for each of the fillable sphere and respective background VOIs were calculated to assess accuracy of measurements, especially in regards to partial volume effects (PVE):

1 $$ \mathrm{RC}\ \left(\%\right)=\frac{A_m}{A_t}\times 100 $$

where *A*_*m*_ is the measured mean activity concentration and *A*_*t*_ is the true activity concentration. The coefficient of variation (COV) of RCs for each hot sphere VOI and day of imaging (8 VOI measurements per COV) was used to quantify both inter- and intra-center variability:

2 $$ {\mathrm{COV}}_{i,n}\left(\%\right)=\frac{\sigma_{i,n}}{\mu_{i,n}}\times 100 $$

where *σ*_*i*, *n*_ is the standard deviation and *μ*_*i*, *n*_ is the mean of RCs for a given sphere size (*i*) and day of imaging (*n*).

Background variability for each sphere size *s* (BV_s_) for the highest activity day of imaging (12 measurements/site × 8 sites = 96 total VOIs) was calculated using

3 $$ {\mathrm{BV}}_s\ \left(\%\right)=\frac{\sigma_s}{\mu_s}\times 100 $$

where *σ*_*s*_ is the standard deviation of the 96 background concentration measurements for a given sphere size *s*, and *μ*_*s*_ is the average of the 96 background concentration measurements for a given sphere size *s*.

Activity in the cold insert (*C*_i_) was quantified using

4 $$ {C}_i\ \left(\%\right)=\frac{A_{m,i}}{A_{t,b}}\times 100 $$

where *A*_*m*, *i*_ is the measured activity concentration in the cold insert, and *A*_*t*, *b*_ is the true background activity concentration.

## Results

**Fig. 2:**
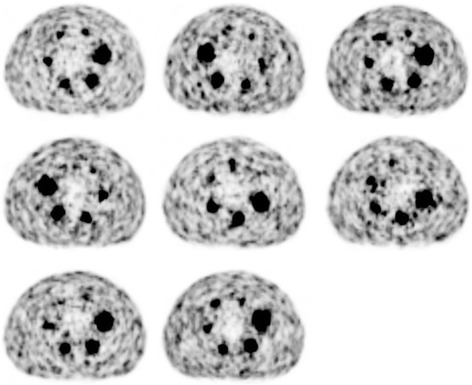
PET images from first day of imaging (~ 3.0 GBq) for each of the eight sites

Activities reported by each site’s dose calibrator were on average 3.62% lower than that reported on the shipping label (median − 4.95%, range − 6.31–0.61%). Figure 2 shows transaxial PET images through the center of the spheres from the first day of imaging for each site.

After visual inspection and manual registration between the attenuation maps and the PET data, adequate registration was obtained in all data sets. Most data sets required approximately a few centimeters translation in the axial direction to properly align the attenuation map with the PET data. At some sites, the phantom was positioned in a reversed direction or the center plate containing the spheres was rotated (Fig. 2), in which case a rotation was required to properly align the attenuation map with the data.

**Fig. 3:**
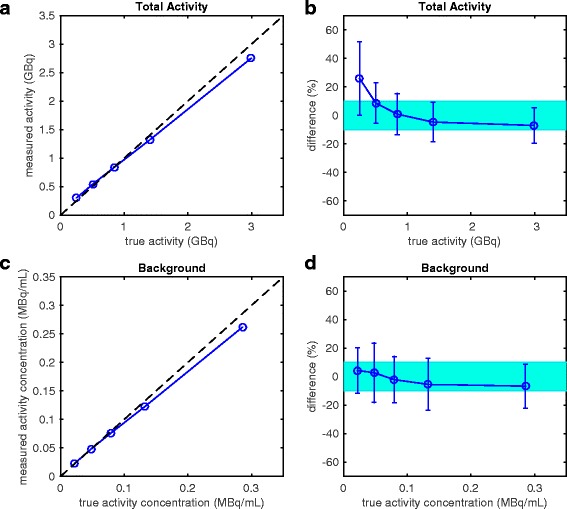
**a** Total measured activity within the FOV compared to the true activity, averaged across the eight sites. The dashed line represents the identity (i.e., where measured equals true). **b** Error in measured activity within the FOV. Each point represents the mean measured activity across the eight sites. Error bars represent one standard deviation across the eight sites’ measurements. The shaded region represents ± 10% error. **c** Mean activity concentration of the 37 mm VOI within the warm background compartment of the phantom compared to the true activity concentration, averaged across the eight sites. The dashed line represents the identity (i.e., where measured equals true). **d** Deviation of measured activity concentration within warm background. Each point represents the mean measured activity concentration of the 37 mm VOI across the eight sites. Error bars represent one standard deviation across the eight sites’ measurements. The shaded region represents ± 10% error

Figure 3a shows the measured activity versus true activity within the whole FOV along the identity line, averaged across the eight sites, and Fig. 3b shows the percent differences in total measured activity and true activity. The total activity measured within the FOV had a median error of − 0.63% across all activity ranges from all sites (8 sites × 5 days of imaging = 40 measurements) (mean 4.71%, range − 23.9–65.0%). All average measurements of total activity were within ± 10% of true activity for activities ≥ 0.5 GBq. The lowest activity measurements (~ 0.3 GBq) overestimated the total activity within the FOV on average by 25.8% (range − 20.8–65.0%).

Figure 3c shows the mean measured versus true activity concentration in the 37 mm VOI in the warm background compartment of the phantom along the identity line, and Fig. 3d shows the percent deviation in this background activity concentration. Mean measured activity concentration had a median error of − 1.68% across all activity ranges from all sites (12 measurements/site × 5 imaging days × 8 sites = 480 measurements) (mean − 1.43%, range − 31.3–32.1%).

**Fig. 4:**
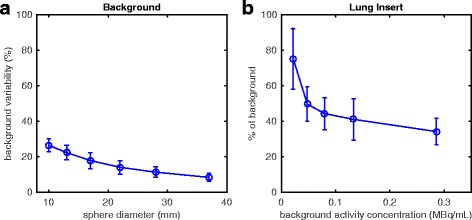
**a** Variability in measurements between 12 × 8 background VOIs for each sphere size. **b** Misplaced counts in cold lung insert reported as percent of true background activity concentration. Each point represents the mean. Error bars represent one standard deviation

Figure 4 shows the noise in the PET imaging volumes, quantified by both the background variability (Eq. 3, Fig. 4a) and the signal intensity in the cold lung insert normalized by the true background activity (Eq. 4, Fig. 4b). The median background variability ranged from 6.48 to 32.2%, measured from the largest and the smallest spheres, respectively, across all sphere sizes (total of 576 warm background VOIs), with a median variability of 16.5% (mean 16.7%). The median cold insert counts (*C*_*i*_) increased from 33.3 to 75.6% in the lung insert from 0.29 MBq/ml to the lower true activity concentrations of 0.02 MBq/ml. The median cold insert counts percentage across 40 PET imaging volumes was 45.3% of the true warm background activity concentration (mean 48.8%, range 23.9–104%). In comparison, the cold insert counts normalized to warm background in the ^18^F phantom image was 26%.

**Fig. 5:**
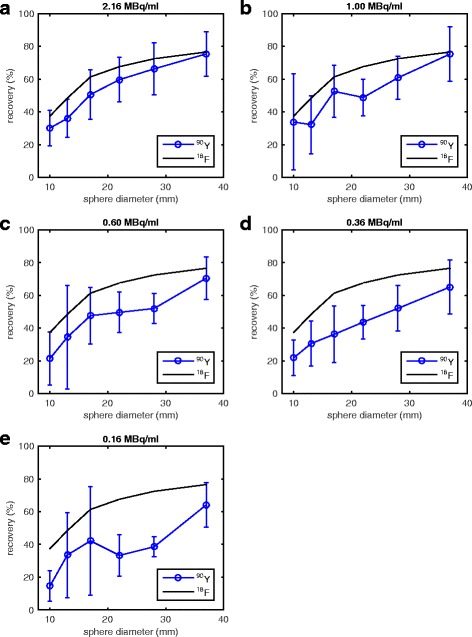
**a**–**e** Recovery coefficients as a function of hot sphere size from five different hot sphere activity concentrations across all sites. Each point represents the mean RC and error bars represent one standard deviation across the eight sites. The COV for each sphere size and hot sphere activity concentrations are shown in Table 3. Measurements from one site’s ^18^F measurements (54.4 MBq) are shown in black

Figure 5 and Table 3 illustrate the RCs and variation of each sphere size averaged over the different institutions. The inter-center variability of imaging with ^90^Y, expressed as the standard deviation on the RC measured at the different sites, is shown on the graphs as the error bars. A comparative measurement done with the same phantom but with high statistics ^18^F is indicated as the solid line on the graphs. The median RC for each hot sphere across all sites and activity concentrations (5 imaging days × 8 sites = 40 measurements per sphere) was 68.7, 52.4, 46.8, 41.8, 27.5, and 22.0% for the 37-, 28-, 22-, 17-, 13-, and 10-mm spheres, respectively. The highest mean RC for any given activity concentration and sphere size was 75.4%, measured on the 37-mm sphere at the highest activity (~ 2.16 MBq/ml). In contrast, the lowest mean RC for any given activity concentration and sphere size was 14.5%, measured from the 10-mm sphere at the lowest activity level (~ 0.16 MBq/ml). Agreement with ^18^F measurements was best for the largest sphere and at the two highest activity levels (1.00 and 2.16 MBq/ml of ^90^Y), with deviation of − 1.7 and − 1.5%, respectively. Variability in measurements increased with decreasing sphere size and decreasing activity.

**Table 3:** Inter-center coefficient of variation (COV) of recovery coefficients (RCs) for each sphere size and activity concentration

	COV (%) (range)				
Sphere diameter (mm)	2.16 MBq/ml	1.00 MBq/ml	0.60 MBq/ml	0.36 MBq/ml	0.16 MBq/ml
10	36.2 (14.0–48.6)	86.6 (6.18–99.8)	75.1 (7.79–51.7)	49.5 (9.83–45.1)	64.1 (2.63–26.9)
13	32.2 (23.5–59.8)	55.1 (18.0–68.3)	92.3 (12.5–112)	44.9 (13.6–49.6)	77.5 (3.47–65.5)
17	29.7 (18.9–69.1)	30.4 (30.3–78.0)	36.4 (27.5–75.8)	47.7 (20.5–75.0)	78.8 (14.7–118)
22	22.7 (41.2–84.1)	22.8 (29.6–63.4)	25.0 (32.6–70.0)	23.7 (30.5–56.1)	37.9 (10.4–49.4)
28	24.1 (42.9–88.1)	21.5 (41.7–80.3)	17.8 (38.0–65.3)	26.8 (26.8–69.6)	15.8 (26.8–46.9)
37	18.0 (56.0–95.0)	22.2 (52.0–103)	18.3 (54.9–92.2)	25.3 (46.4–91.4)	21.1 (45.2–81.3)

Values in parentheses represent the range of RCs (min–max)

**Fig. 6:**
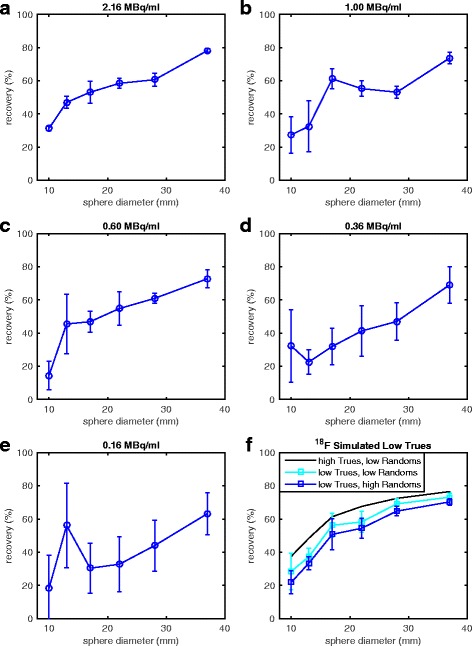
**a**–**e** Recovery coefficients as a function of hot sphere size from three consecutive scans at a single site. Each point represents the mean RC, and error bars represent one standard deviation. The COV for each sphere size and hot sphere activity concentrations are shown in Table 4. **f** Measurements from raw ^18^F data (black line), ^18^F data with similar ^90^Y true count statistics (light blue square), and ^18^F data with similar true count statistics and randoms fraction to ^90^Y. Again, each point represents the mean RC and error bars represent one standard deviation across the ten 3-s ^18^F datasets

Figure 6a–e and Table 4 illustrate the RC variability obtained from one site (Norwegian University of Science and Technology, Trondheim, Norway) who acquired three consecutive scans for each imaging day. The median RC for each hot sphere across the three scans and all activity concentrations (5 imaging days × 3 scans = 15 measurements per sphere) was 74.8, 56.9, 55.6, 47.2, 44.1, and 24.3% for the 37-, 28-, 22-, 17-, 13-, and 10-mm spheres, respectively. . At the highest activity level, variability between scans for all hot spheres was < 13% (mean 6.24%, median 5.77%, range 1.42–12.4%). All spheres ≥ 17 mm with ≥ 0.60 MBq/ml had < 20% variability in their RCs. The 37-mm sphere had a variability in measurements ≤ 20% for all activity levels. Again, variability increased with decreasing sphere size and decreasing activity. Table 5 lists the COV and range for these scans for RC of total activity and background activity concentration and *C*_*i*_ values of the cold lung insert. Variability tended to increase with decreasing activity for these VOIs as well, with the exception of total activity, which tended to remain rather stable. Figure 6f shows measurements from the ^18^F phantom, reconstructed into smaller data sets with both trues counts statistics and/or randoms fraction typically seen with ^90^Y PET data from the highest activity imaging day. Total trues for these smaller ^18^F data sets were 2.07 × 10^6^ and those reported by one site for ^90^Y were 1.54 × 10^6^. The randoms fraction for typical ^18^F datasets was 31% and that for the simulated high-randoms was 77%. The randoms fraction for ^90^Y was approximately 75%.

**Table 4:** Intra-center coefficient of variation (COV) of recovery coefficients (RCs) for each sphere size and activity concentration

	COV (%) (range)				
Sphere diameter (mm)	2.32 MBq/ml	1.06 MBq/ml	0.65 MBq/ml	0.38 MBq/ml	0.17 MBq/ml
10	4.49 (29.7–32.3)	40.2 (20.8–40.2)	59.4 (8.71–24.3)	67.6 (14.4–56.6)	108 (4.42–40.9)
13	7.58 (44.1–51.0)	47.1 (22.6–50.2)	39.7 (27.2–63.3)	32.9 (16.6–30.9)	45.3 (29.1–79.6)
17	12.4 (48.8–60.6)	9.79 (57.5–68.1)	13.4 (40.4–53.0)	34.5 (21.6–43.5)	49.6 (15.6–45.7)
22	5.06 (55.6–61.5)	8.35 (50.2–59.2)	18.2 (43.4–62.0)	36.8 (27.8–57.7)	50.8 (14.5–47.1)
28	6.48 (56.2–63.7)	6.69 (49.9–57.0)	4.89 (58.9–64.3)	23.9 (34.9–56.9)	35.0 (27.2–57.4)
37	1.43 (77.0–79.0)	4.79 (70.1–77.1)	7.52 (66.6–77.0)	16.0 (56.6–77.4)	20.0 (53.9–77.7)

Values in parentheses represent the range of RCs (min–max)

**Table 5:** Intra-center coefficient of variation (COV) between three consecutive scans of recovery coefficients (RCs) of total activity and background activity concentration and of counts in the cold lung insert (*C*_*i*_) on each imaging day

	COV (%) (range)				
VOI	3.0 GBq	1.4 GBq	0.8 GBq	0.5 GBq	0.2 GBq
Total activity	0.80 (96.7–98.2)	0.47 (102–102)	0.78 (106–107)	0.79 (114–115)	0.80 (139–141)
Background	16.8 (46.5–147)	26.9 (33.6–209)	27.9 (32.8–179)	34.6 (32.8–254)	50.4 (30.5–423)
Lung insert	4.51 (31.5–34.5)	5.99 (39.6–44.6)	2.82 (37.4–39.1)	5.05 (39.9–44.5)	10.2 (61.1–74.1)

Numbers in parentheses represent the range of RCs for total activity and background activity concentration and *C*_*i*_ for the lung insert (min–max)

## Discussion

Despite the low positron yield from ^90^Y, PET imaging of this isotope has proven both feasible and useful in previous literature [11–13, 15–17, 23, 24] and is further demonstrated in this work. The results from this study and that reported by Willowson et al. in the PET/CT QUEST study [20] also demonstrate the feasibility of performing multi-institutional clinical studies focused on ^90^Y PET-based dosimetry.

Even though this multi-institutional phantom study with ^90^Y PET imaging has been performed previously on PET/CT [20], there are several key differences between Siemens Biograph PET/CT scanners and Siemens Biograph mMR (PET/MRI) scanners that motivate the need to perform this study on PET/MRI. First, the Biograph mMR utilizes avalanche photodiode (APD) detectors instead of typically used photomultiplier tubes (PMT), to allow for compatibility with the MR magnetic field. However, this comes at the cost of not having TOF capabilities. Additionally, compared to the Biograph PET/CT scanners, the mMR exhibits increased sensitivity due to the geometrical arrangement of the detectors: 15.0 kcps/MBq) [25] versus 8.1 kcps/MBq on the Biograph 40 (non-TOF) PET/CT [26]. As discussed previously, the difference in sensitivities is due to a longer axial FOV and shorter ring diameter on the Biograph mMR [23]. The tight geometry and wider coincidence timing window in mMR result in higher random rates, but the smaller block size results in lower singles rate per detector block in comparison to Siemens PET/CT scanners [23]. All of these factors (i.e., difference in electronics and higher sensitivity from a different geometry), combined with low annihilation counts and high randoms rates from Bremsstrahlung radiation, make the similarity of the convergence properties of OSEM imaging reconstruction different between Biograph PET/CT and Biograph PET/MRI scanners. This was demonstrated in our previous phantom study, where we found that the optimal reconstruction parameters, using the same number of subsets, post-reconstruction filter size, and resolution recovery, were at three iterations on the mMR instead of two iterations for Biograph PET/CT scanners, as suggested by Willowson et al. [20]. Therefore, it is necessary to demonstrate the quantitative accuracy and test the variability between scanners on mMR, especially since this has not been done.

^90^Y has high randoms rates (due to a high flux of Bremsstrahlung radiation) and low trues statistics (due to low annihilation counts); therefore, a careful handling of the randoms in the reconstruction proves essential in iterative reconstruction algorithms. In previous generations of OSEM, the pre-subtraction of randoms and scatter results in significant bias [27, 28]. Siemens mMR uses 3D OP-OSEM with PROMPTS+RANDOMS data acquisition, as done in Biograph mMR and mCT, there is no pre-subtraction of scatter or randoms estimates, and therefore the algorithm bias is reduced [29]. However, in situations of very low counts such with ^90^Y imaging, the convergence properties of the algorithm may be such that more iterations are needed at the expense of increased noise. In our phantom evaluation [23], we limited the noise in the images by stopping after three 3D OP-OSEM iterations (with 21 subsets resulting in 63 updates) and applying a 5 mm Gaussian post-reconstruction filter. As shown in [23], images reconstructed with more iterations and sharper filter resulted in unacceptably noisy images. The low count statistics and high randoms fraction for ^90^Y PET imaging are likely the cause of lower count recovery compared to higher statistics ^18^F PET imaging. Evidence of this is shown in Fig. 6f. Optimized image reconstruction algorithms, with extremely low count statistics and high random counts, have yet to be resolved [20, 23, 30]. This is an area of future research.

Recovery of the total activity within the whole FOV was consistent among sites, with most measurements within ± 10% of the true values, especially above 0.5 GBq. These values are consistent with those reported by Willowson et al. in the PET/CT QUEST study for 19 Siemens Biograph TOF PET/CT scanners [20], though our reported standard deviation on the Biograph mMR at 0.5 GBq was slightly higher, likely due to a smaller number of sites. Our total activity results were closer in agreement with the expected activity than those reported for Siemens Biograph non-TOF PET/CT scanners in the same QUEST study, where mean error in the FOV at 0.5 GBq was approximately + 20% and standard deviation in this error was approximately ± 60% [20]. Our study performed an extra scan at a lower activity level (0.3 GBq) that was not performed in the PET/CT QUEST study. The extra scan at a lower activity level represents the subset of patients who are administered the lowest activity available for resin microsphere treatment. Total activity measurements in this range were, on average, overestimated by > 20%, with a standard deviation reaching beyond ± 20%. This trend in overestimation of total activity with decreasing activity has been reported in previous studies using both TOF and non-TOF Siemens Biograph PET/CT scanners [20, 24]. A possible explanation could be extremely low count statistics resulting in higher noise, uncertainty in scatter correction, or artificial peaks in the data resulting in measured activity higher than the true activity. This effect may be less prominent in TOF reconstructions, since it is known that TOF reduces noise by reducing noise propagation in both forward- and back-projections at each iteration [31].

Activity measurements in the warm background compartment of the phantom were excellent, with the mean error consistently < 10%. These values are consistent with those reported in the QUEST study for both the Siemens Biograph TOF (> 1 iteration, no post-reconstruction filter reconstructions only) and non-TOF PET/CT (PROMPTS+RANDOMS reconstructions only) scanners [20]. The standard deviations in our warm background measurements were also comparable to those reported in the QUEST PET/CT study. It is important to note that the background compartment of the phantom was intended to simulate the non-zero uptake of normal liver parenchyma, as is commonly seen in clinical studies [15, 16]. Even though liver tumors tend to take up higher concentrations of the ^90^Y microspheres, which is the goal of radioembolization, the normal liver parenchyma often takes up some of the microspheres as well, albeit usually at a lower concentration. Thus, it was imperative to include warm background activity in the large compartment of the phantom in an attempt to simulate the clinical setting. In our experience, the ratio of tumor:normal liver (“background”) activity concentrations varies from patient to patient. Future work includes investigating the effects of tumor:background activity concentrations on image quality.

Noise in the PET imaging volumes was quantified by both the background variability on the high activity day of imaging (~ 0.29 MBq/ml) (Eq. 3) and the scatter/background counts in the cold (no activity) lung insert of the phantom (Eq. 4). Background variability on the Biograph mMR was significantly lower than that reported for both the TOF and non-TOF Biograph PET/CT scanners in the QUEST study (16.7% mean versus ~ 30, 50, and 38% means for TOF and Gaussian post-reconstruction filter, TOF and no post-reconstruction filter, and non-TOF reconstructions, respectively) [20]. However, scatter counts in the lung insert from the mMR (49.0% mean) were higher than those from Biograph TOF PET/CT scanners (~ 30% mean) and non-TOF PET/CT scanners using PROMPTS+RANDOMS mode (~ 35% mean). They were, however, lower than those from Biograph non-TOF PET/CT scanners in NETTRUES mode (~ 60% mean) [20]. A possible reason for the higher rate of scatter counts in the cold lung insert compared to those reported by Willowson et al. could be due to fact that the attenuation maps from this study were vendor-provided, since attenuation maps of phantoms cannot be directly measured with MRI. Depending on the type of lung insert used in the vendor provided maps (styrofoam or solid), this could affect the attenuation properties and scatter estimates used in the reconstruction. However, private communication with a representative from Siemens confirmed that the vendor-provided attenuation map was measured from identical phantoms used in this study. Another possibility is that the difference in geometry of the Biograph mMR scanner (i.e., longer axial FOV) may allow for more scatter counts than the Biograph mCT or non-TOF PET/CT scanners. Regardless, scatter counts are higher for any of these three scanners compared to ^18^F when imaging ^90^Y. A possible explanation is that in very low count studies, the estimated scatter distribution may be inaccurate. The scatter distribution is iteratively estimated from the measured activity distribution and the phantom attenuation map. When count statistics are low in an imaging dataset, residual scatter counts may remain in regions of cold (i.e., no) activity, and the data supports this theory. For the low activity scan, the counts in the cold insert were approximately 76% of warm background, which is significantly higher than that measured for ^18^F at 26%. At the highest ^90^Y activity scan, cold insert counts was approximately 33% of warm background, much closer to that of ^18^F. Higher residual scatter counts in cold areas may also explain why hot sphere RCs for low total activity scans are much lower that for ^18^F. Others have investigated incomplete convergence in these cold areas [32], and this remains an area of active research.

Count recovery in the higher activity concentrated (hot) spheres was good, with mean RCs ranging from approximately 30–75% on the highest activity imaging day (~ 2.16 MBq/ml). These values are consistent with those from a previous ACR phantom study at two of the institutions included in this study [23]. They are also consistent with those reported for the same total activity level in the QUEST study for the Siemens Biograph TOF PET/CT scanners (two iterations, 5-mm Gaussian post-reconstruction filter) and better than those for the non-TOF PET/CT scanners (all reconstructions) [20]. RCs from ^90^Y PET imaging were also lower than those from ^18^F PET imaging, with the exception of the largest sphere size at the highest activity. We report lower ^18^F RCs than Willowson et al., who reported RCs approaching near 100% for the largest sphere [20]. Discrepancy in these measurements is likely due to a difference in the method for drawing VOIs: they used a region-growing approach, where VOIs were drawn at 50% of the maximum value, whereas we used the known sphere diameter to draw VOIs. Using an attenuation map-based method for drawing VOIs, as opposed to a region-growing approach, is known to decrease RCs in PET images [24]. Our method is more susceptible to partial volume effects but is more representative of what is performed for individual lesion dosimetry. As is characteristic of PET imaging studies, whether using ^90^Y or a standard isotope-like ^18^F, RCs degraded with decreasing VOI size [21, 23, 24, 33, 34]. Combining this with the high noise, which is characteristic of ^90^Y PET images, it is apparent from Fig. 2 that very small regions of high uptake (i.e., the smallest sphere) are difficult, if not impossible, to identify. This is a limitation that must be considered in clinical applications of ^90^Y PET imaging when assessing microsphere uptake in small lesions. RCs of any given hot sphere size also slightly decreased with decreasing activity concentration, similar to previous phantom studies [20, 23]. Contributing factors to sub-optimal recovery, especially below ^18^F, could include partial volume effects, low positron statistics, and high randoms rates. Low annihilation counts, and thus low trues rates, especially combined with high randoms rates, are limiting factors of OSEM reconstruction algorithms, as previously discussed. Low trues rates are demonstrated as a contributing factor for poorer recovery in Fig. 6f for even a standard isotope such as ^18^F. However, this does not account for the entire discrepancy between these two isotopes. Handling these combined factors in iterative reconstruction algorithms is an active area of research, not just in ^90^Y PET imaging [30] but also in gated-cardiac PET imaging where statistics are often low [35].

Inter-center variability, as quantified by the COV for each sphere size and activity concentration, was acceptable (< 25%) for sphere diameters ≥ 22 mm and ≥ 0.60 MBq/ml. For the highest activity level, inter-center variability was < 23% for sphere diameters ≥ 22 mm. In the multi-institutional phantom study by Fahey et al., nine sites tested the variability of imaging ^18^F on PET/CT using an ACR phantom in preparation for a multi-institutional clinical trial. They reported COVs in RCs of 5.9, 21.2, and 17.0% for VOI diameters 25, 16, and 12 mm, respectively [19]. Though these values are lower than what we measured for our comparably sized sphere VOIs, considering the noisy nature of ^90^Y PET images, our results offer promise for the ability to reliably perform multi-institutional clinical studies of ^90^Y PET-based dosimetry with the Siemens Biograph mMR.

Intra-center variability was also acceptable, < 7% for sphere diameters ≥ 22 mm, which was lower than that reported by the QUEST study for the Biograph TOF PET/CT (5, 4, and 8% for sphere diameters 37, 28, and 22 mm, respectively) on the highest activity imaging day (2.32 MBq/ml) [20]. Yet, for low activity concentrations, this variability increased significantly, and COVs of the spheres became comparable to those seen at a multi-institutional level, suggesting that inter-center variation at low activities is likely more due to scanner performance than experimental error. Conversely, the large variability seen at the inter-center level at even the highest activity concentration (Fig. 5a–c) that is generally not present at the intra-center level for these same activity concentration ranges (Fig. 6a–c) suggests there could be discrepancies in the phantom preparation in addition to differences between individual scanner performance.

Several limitations exist with this study. As mentioned previously, we used a vendor-provided attenuation map since direct attenuation map acquisition of phantoms is not accurate on PET/MRI scanners. A study by Ziegler et al. found that the MR-derived attenuation map of the NEMA image quality phantom, obtained from the Siemens Biograph mMR PET/MRI scanner, only captured the photon attenuation of water inside the phantom but failed to capture the plastic housing of the phantom itself, which significantly degraded both the quantitative accuracy and background variability of the PET images. Image quality was improved when a CT-derived attenuation map of the phantom was used instead [21]. It is important to note that this necessity for using a CT-derived attenuation map only applies to phantom studies and is not necessary for clinical studies. Since the attenuation maps were not measured directly from the PET/MRI scanner at the time of PET imaging, they had to be manually registered and verified to the PET volumes in order to incorporate into offline reconstruction. Manual registration of the attenuation map to the PET data may have introduced error during the reconstruction procedure since it is not an exact process; however, visual inspection confirmed satisfactory registration between the data sets. We attempted to further mitigate any re-positioning errors both at the intra- and inter-center level by using a phantom cradle and positioning device to replicate phantom placement in the scanner between sites. Furthermore, since we were unable to image the phantom’s plastic housing directly with MRI, we could not see if the spheres were filled completely; thus, the “true” activities may have actually been overestimates of what was actually filled in the spheres. Since our RCs agreed well with those reported in the QUEST study by Willowson et al. for Siemens Biograph TOF PET/CT scanners, we consider this effect to be negligible. Lastly, a general limitation of PET/MR imaging is the lack of attenuation correction for coils, which may degrade quantitative assessment of the PET images in clinical studies. Since we are assessing only the PET camera performance in this study and not the MR-based attenuation correction algorithm, we excluded coils from this study. However, this is something to consider in clinical cases when coils are often used.

PET/MRI scanners from other vendors, such as GE, were not included in this study due to a lack of other vendor sites at the time of conducting this study. Future work will test the performance of GE PET/MRI scanners.

## Conclusions

^90^Y PET measurements from Siemens Biograph mMR (PET/MRI) scanners are acceptable and reproducible at the multi-institutional level. This study may provide insight into the minimum activity concentrations (≥ 0.60 MBq/ml) and VOI size (≥ 22 mm diameter) for accurate and reproducible measurements across institutions. Performance is comparable to that of its TOF PET/CT counterpart and may suggest that multi-institutional clinical studies of ^90^Y PET-based dosimetry using Siemens hybrid PET scanners can include both PET/MRI and TOF PET/CT scanners, although MRI may offer additional advantages, such as superior soft-tissue contrast for easy delineation of liver lesions.

## Abbreviations

^18^F: Fluorine-18
3D: Three-dimensional
^90^Y: Yttrium-90
APD: Avalanche photodiode
COV: Coefficient of variation
CT: Computed tomography
DTPA: Diethylenetriaminepentaacetic acid
EDTA: Ethylenediaminetetraacetic acid
FOV: Field of view
IQ: Image quality
MRI: Magnetic resonance imaging
NEMA: National Electrical Manufacturers Association
OP-OSEM: Ordinary poisson ordered subset expectation maximization
PET: Positron-emission tomography
PSF: Point spread function
PVE: Partial volume effect
QUEST: Quantitative uptake evaluation in SIR-spheres therapy
RC: Recovery coefficient
SPECT: Single photon emission computed tomography
TOF: Time of flight
VOI: Volume of interest
